# The interplay of the microbiome, host genetics, and epigenetic modifications in gastric cancer

**DOI:** 10.3389/fmicb.2026.1834439

**Published:** 2026-07-22

**Authors:** Ting Zhou, Guona Li, Wujie Ye, Luyi Wu, Huirong Liu, Jing Guo, Yunfan Wen, Jing Li, Mengdie Wu, Weiwei Li, Huangan Wu

**Affiliations:** 1 Yueyang Hospital of Integrated Traditional Chinese and Western Medicine, Shanghai University of Traditional Chinese Medicine, Shanghai, China; 2 Shanghai Research Institute of Acupuncture and Meridian, Shanghai, China; 3 Nanjing University of Chinese Medicine, Nanjing, China; 4 School of Traditional Chinese Medicine, Shanghai University of Traditional Chinese Medicine, Shanghai, China

**Keywords:** cancer treatments, DNA methylation, gastric carcinogenesis, genetic polymorphism, microbial metabolites

## Abstract

Gastric cancer is one of the most prevalent gastrointestinal malignancies worldwide, with *Helicobacter pylori* infection, host genetic susceptibility and environmental exposure serving as major driving risk factors. Accumulating studies have demonstrated that host genetics, the microbiome and epigenetic modifications collectively govern gastric cancer initiation and progression. These three components form a bidirectional regulatory axis: host genetic profiles and epigenetic remodeling shape the composition of endogenous microbial communities, while the microbiome and its metabolites trigger epigenetic reprogramming to modulate transcription of oncogenes and tumor suppressors. Deciphering this intricate tripartite regulatory network holds great potential to facilitate the development of precise therapeutic interventions for gastric cancer. This review delineates the respective roles of host genetics, the microbiome and epigenetic modifications throughout gastric cancer evolution and summarizes corresponding prospective intervention strategies. We elaborate on the reciprocal interplay between the microbiome and host genetic/epigenetic factors, and highlight the vital clinical significance of this crosstalk for gastric cancer prevention and treatment.

## 1 Introduction

Gastric cancer is the fifth most common malignancy worldwide and a leading cause of cancer-related mortality. In 2022, nearly 1 million new cases were reported globally, resulting in more than 650,000 deaths (Bray et al., 2024). The development of gastric cancer is a complex process influenced by multiple factors, including infections, high-salt diets, smoking, alcohol consumption, and genetic susceptibility (Wang Y. et al., 2025; Liu et al., 2026). *Helicobacter pylori (H. pylori)* is internationally recognized as a Group I carcinogen for gastric cancer and infects approximately half of the world’s population. A strong spatial correlation exists between *H. pylori* infection and gastric cancer incidence, making it a key driver of the disease (de Martel et al., 2020). Unhealthy lifestyle factors, such as high-salt diets, smoking, and alcohol consumption, can damage the gastric mucosal barrier and increase the risk of gastric cancer (Qin et al., 2025). Moreover, Epstein–Barr virus (EBV) infection is associated with approximately 9% of gastric cancer cases, while genetic susceptibility plays a major role in certain familial cases (Guerra et al., 2025; Shi et al., 2026). Effective management of these risk factors can reduce the incidence of gastric cancer (Smyth et al., 2020). In recent years, the overall incidence of *H. pylori*-associated gastric cancer has declined (Anderson et al., 2018). Nevertheless, the incidence of gastric cancer among young adults in high-income countries has increased rather than decreased (Arnold et al., 2020; Park et al., 2025). This epidemiological trend suggests that additional, yet unelucidated, factors and mechanisms are involved in the development and progression of gastric cancer.

**Figure 1:**
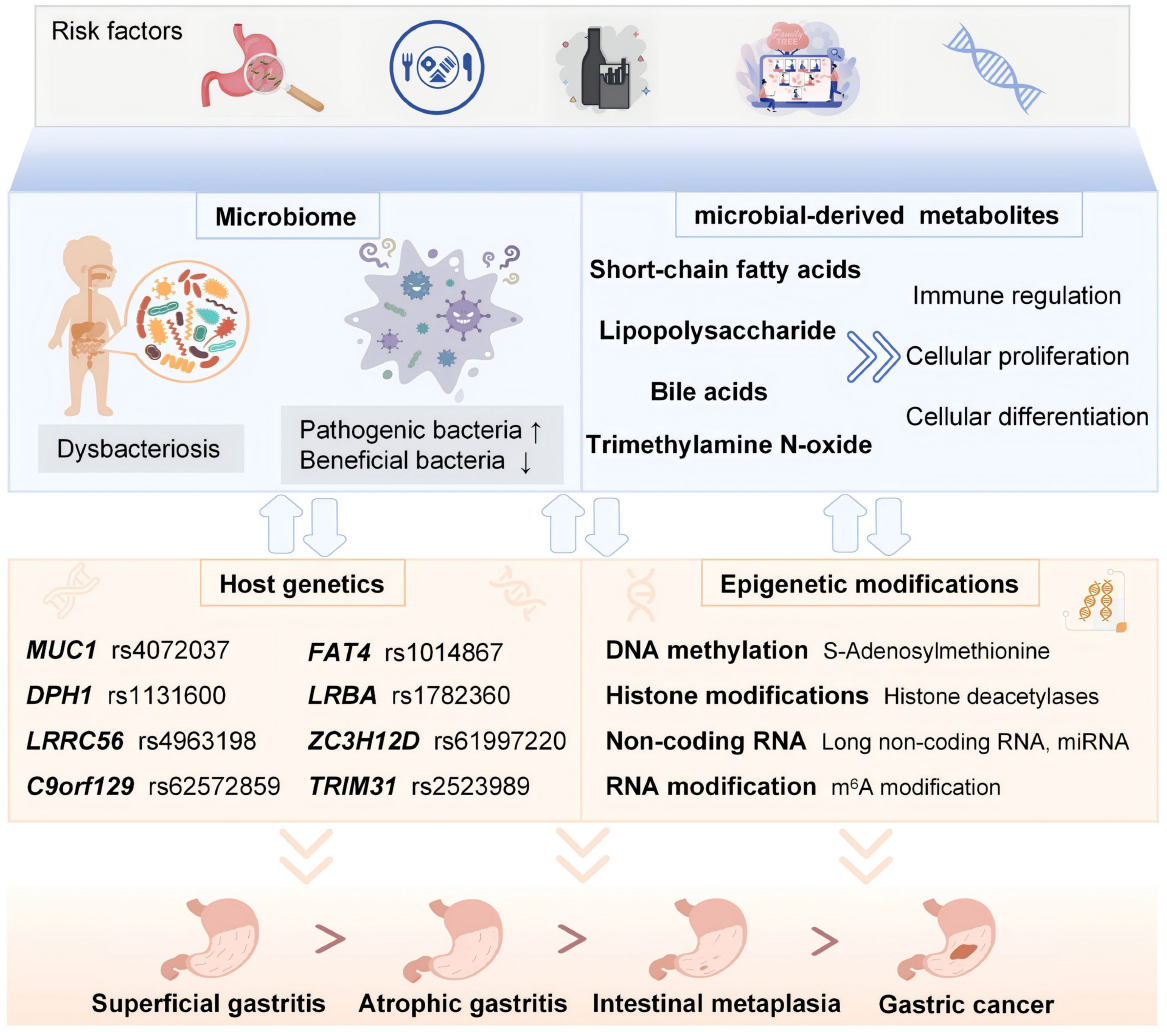
The interplay between the microbiome, microbial metabolites, and host genetics and epigenetics in gastric cancer. Exposure to various risk factors induces dysbiosis of the human microbiota, consequently modulating the profile of microbial metabolites. The resultant changes in microbial and metabolic signatures can transcriptionally influence host epigenetic mechanisms, while the host genetics concurrently defines the architecture and function of the microbial community. The interplay of these factors ultimately leads to gastric mucosal injury, persistent inflammation, and progression to gastric cancer. Created with Figdraw.

The microbiome has emerged as an important factor that has attracted considerable attention in recent years (Zhang L. et al., 2025). The discovery of *H. pylori* overturned the conventional view that microorganisms cannot colonize the highly acidic gastric environment (Bessede and Megraud, 2022). Accumulating evidence indicates that dysbiosis of the gastrointestinal microbiota contributes to gastric tumorigenesis. Under physiological conditions, the microbiota maintains microenvironmental homeostasis through dynamic interactions with the host immune and metabolic systems (Li Y. et al., 2022). Once the microbial community structure is disrupted or microbial metabolites are altered, gastric mucosal injury and aberrant immune responses may be directly induced (Wang S. et al., 2022). Furthermore, the microbiota regulates host gene expression through epigenetic mechanisms, alters the biological phenotypes of epithelial cells, and ultimately mediates oncogenic transformation (Sayed et al., 2020; Wang L. et al., 2022; Baral et al., 2024). Conversely, host genetic polymorphisms and aberrant epigenetic modifications can disrupt microbial homeostasis and accelerate disease progression (Alenghat et al., 2013; Coker et al., 2018). This complex interplay may influence the occurrence and progression of gastric cancer (Figure 1). Clarifying the bidirectional relationships among the microbiome, host genetics, and epigenetic modifications will further elucidate the impact of the microbiota on host health (Pepke et al., 2024; Wang C. et al., 2025).

However, most existing reviews focus only on the unidirectional relationships among the microbiome, host genetics, and epigenetic modifications in gastric cancer and lack a systematic synthesis of their integrated bidirectional regulatory networks. In addition, the clinical translational potential of microbiota-targeted therapies based on these regulatory networks has not been fully explored. Accordingly, this review systematically examines the interactions among the microbiome, host genetics, and epigenetic modifications during gastric cancer development and further explores novel therapeutic strategies with potential clinical applications.

## 2 The microbiome and its metabolites in gastric cancer

### 2.1 The microbiome in gastric cancer

The occurrence and progression of gastric cancer are closely associated with alterations in microbial composition and their metabolites. Microbial dysbiosis exhibits distinct characteristics across the gastric mucosa, gut, and oral cavity. High-throughput sequencing studies have revealed a progressive decline in both *α*-diversity and *β*-diversity of the gastric microbiota as the disease advances from a healthy state through precancerous lesions to full-blown gastric cancer (Ferreira et al., 2018; Liu et al., 2019; Wang C. et al., 2023). A cohort study of Mongolian people based on 16S rRNA sequencing found that, healthy people exhibited the highest bacterial *α*-diversity, followed by patients with intestinal metaplasia and gastric cancer. In contrast, patients with superficial gastritis and atrophic gastritis displayed the lowest diversity. Simultaneously, several bacterial genera—including *Enterococcus*, *Lactobacillus*, *Carnobacterium*, *Glutamicibacter*, *Fusobacterium*, *Paeniglutamicibacter*, and *Parvimonas*—were significantly enriched in gastric cancer tissues (Gantuya et al., 2020). Another study reported marked reductions in both α-diversity and *β*-diversity in gastric cancer patients compared with those with chronic gastritis. Although the dominant phyla—*Proteobacteria*, *Firmicutes*, *Bacteroidetes*, *Actinobacteria*, and *Fusobacteria*—were consistent between groups, gastric cancer patients exhibited higher proportions of *Actinobacteria* and *Firmicutes* and lower proportions of *Bacteroidetes* and *Fusobacteria*, further supporting a strong association between microbial dysbiosis and gastric cancer development (Ferreira et al., 2018).

Alterations in the gut microbiota have also been linked to gastric cancer risk. Studies indicate that patients with gastric cancer exhibit increased intestinal abundance of *Enterobacteriaceae* and decreased abundance of *Bacteroidaceae* (Liang et al., 2019). Enrichment of *Enterobacteriaceae* is closely associated with intestinal inflammation and metabolic reprogramming, which may facilitate microbial adaptation and contribute to tumor progression (Kitamoto et al., 2020). Notably, *Enterobacteriaceae* enrichment has been confirmed as a common feature across multiple gastric cancer subtypes and is significantly associated with increased risk of gastrointestinal mortality (Salosensaari et al., 2021; Sarhadi et al., 2021). Furthermore, a large-scale cohort study in China demonstrated that fecal *Streptococcus anginosus* may serve as a potential biomarker for early gastric cancer screening (Zhou C. B. et al., 2022).

As the second largest microbial reservoir in the human body, the oral microbiota also plays a significant role in gastric cancer development. Microbial translocation from the oral cavity to the gastrointestinal tract can influence the microbial ecosystems of the oral cavity, stomach, and intestine, thereby participating in both physiological and pathological processes (Park et al., 2021). Compared with the precancerous stage, the oral microbial profiles of gastric cancer patients show significant changes. Specific taxa, including *Porphyromonas stomatis*, *Streptococcus exigua*, *Parvimonas micra*, *Streptococcus anginosus*, and *Dialister pneumsintes*, have been proposed as biomarkers distinguishing gastric cancer from precancerous lesions (Coker et al., 2018). Salivary microbiota analyses have further revealed significant enrichment of *Streptococcus* and *unclassified Streptophyta* in gastric cancer patients, accompanied by reductions in *Fusobacterium*, *Haemophilus*, *Neisseria*, *Parvimonas*, and *Prevotella* (Huang et al., 2021). These dysregulated oral microbes may indirectly drive the progression of gastric cancer by promoting chronic inflammation and accumulating carcinogenic nitrosamines.

The above studies indicate that dysbiosis of the gastrointestinal and oral microbiota is closely associated with the occurrence and progression of gastric cancer. It is worth noting that these studies are primarily based on 16S rRNA sequencing. This method can only characterize differences in microbial composition and provide a preliminary assessment of the association between alterations in microbial structure and gastric cancer; however, it is insufficient to elucidate the specific molecular mechanisms through which microbes affect the host epigenome or to distinguish causal relationships between microbial alterations and disease progression. In addition, differences in sample handling and analytical procedures across studies further complicate direct comparisons. In the future, multi-omics integration strategies combined with functional validation experiments will be required further to decode the microbe–host epigenetic regulatory network, thereby overcoming the limitations of current single-omics sequencing approaches.

### 2.2 Microbial metabolites in gastric cancer

Beyond microbial composition, microbial metabolites play pivotal roles in gastric carcinogenesis. Increasing evidence indicates that metabolites such as bile acids (BAs), lipopolysaccharide (LPS), short-chain fatty acids (SCFAs), and trimethylamine N-oxide (TMAO) influence immune regulation, cellular proliferation, and differentiation, thereby contributing to gastric cancer development (Wang S. et al., 2022; Stonans et al., 2023). BAs may reflux into the stomach, promoting intestinal metaplasia, while simultaneously activating the protein kinase C/cyclooxygenase-2 signaling pathway, inducing apoptosis in gastric mucosal cells and promoting tumor invasion (Shi et al., 2016; Wu et al., 2018; Yu et al., 2019; Qu and Shi, 2022). Their synergistic interaction with LPS further upregulates the IL-6/JAK1/STAT3 signaling pathway, amplifying inflammatory responses and promoting the progression of gastric cancer (Wang S. et al., 2022).

Although SCFAs have been associated with anti-inflammatory effects in some studies, their abnormal accumulation in the stomach may also contribute to carcinogenic mechanisms (Nouri et al., 2023). In addition, TMAO—a metabolite derived from dietary choline—has been shown to alter gene expression in *H. pylori*–induced gastric epithelial cells, thereby enhancing inflammatory signaling. Elevated TMAO levels are positively associated with gastric cancer risk in males and play a significant role in tumor invasion and metastasis (Wu et al., 2017; Stonans et al., 2023). Moreover, microbiota-mediated ethanol oxidation exposes the gastric mucosa to increased acetaldehyde concentrations, further elevating carcinogenic risk (Salaspuro, 2011; Maejima et al., 2015). Systematic investigation of these microbial metabolic pathways may provide novel insights into early prevention strategies and targeted therapeutic interventions for gastric cancer.

Dietary patterns, antibiotic exposure, and geographic variation represent important confounding factors when investigating associations among the microbiota, microbial metabolites, and gastric cancer. Diet serves as a critical regulator of microbial communities and shapes the production of microbial metabolites (Hernandez-Valles et al., 2026). In gastric cancer, diet-induced shifts in microbial profiles may be erroneously attributed to tumorigenesis. Long-term antibiotic exposure can markedly disrupt the structural balance of the microbiota (Olivares et al., 2025). Patients with gastric cancer frequently receive antibiotics to alleviate gastrointestinal symptoms, suggesting that observed microbial differences may partially result from pharmaceutical intervention rather than underlying pathological lesions. In addition, individuals from different geographic regions exhibit distinct baseline microbiota compositions, which may limit the generalizability of research findings across diverse populations (Porras et al., 2021). Adjusting for these confounding factors facilitates the more accurate identification of microbiota- and metabolite-related alterations specifically associated with gastric cancer.

## 3 Genetic and epigenetic modifications in gastric cancer

### 3.1 Host genetics

The host genetic background constitutes a critical intrinsic driver of gastric carcinogenesis. Up to 15% of gastric cancer cases exhibit familial clustering, and approximately 10% of patients carry pathogenic germline variants (PGVs) (Lott and Carvajal-Carmona, 2018). Genome-wide association studies (GWAS) and whole-exome sequencing have identified a panel of gastric cancer susceptibility genes, including Tumor Protein p53 (*TP53*), Cadherin 1 (*CDH1*), Catenin Alpha 1 (*CTNNA1*), Ataxia Telangiectasia Mutated (*ATM*), Breast Cancer Susceptibility Genes 1 and 2 (*BRCA1/2*), and MutS Homolog 2 (*MSH2*). These genes primarily participate in the regulation of cell adhesion, oncogenic signaling pathways, and DNA damage repair, collectively forming a regulatory network governing genetic susceptibility to gastric cancer (Ji et al., 2020; Herrera-Pariente et al., 2021; Usui et al., 2023; Li et al., 2026). When evaluated comprehensively in terms of mutation penetrance and clinical translational value, these genes contribute differently to gastric tumorigenesis. *TP53*, *CDH1*, and *CTNNA1* exhibit relatively high mutation penetrance (Marroquin-Estrada et al., 2025; Lobo et al., 2026). In contrast, *ATM*, *BRCA1/2*, *MSH2*, and most single-nucleotide polymorphisms (SNPs) identified through GWAS display relatively low penetrance. Patients with gastric cancer carry significantly higher burdens of PGVs in these susceptibility genes than the general population (Gilad et al., 2025).

*TP53* is a master tumor suppressor gene that encodes the p53 protein, which orchestrates cell-cycle arrest, apoptosis, and DNA damage repair (Wang H. et al., 2023). *TP53* mutations represent the most prevalent somatic genetic alterations in gastric malignancies (Kemp et al., 2026). Such mutations not only abolish canonical tumor-suppressive functions but also rewire intracellular signaling pathways to facilitate tumor invasion and metastasis (Huber et al., 2024). Pharmacological strategies that selectively induce senescence in *TP53*-mutant gastric cancer cells have demonstrated potent antitumor efficacy in preclinical models (Wang K. et al., 2023). Epithelial polarity and tissue architectural integrity are coordinately maintained by *CDH1* and *CTNNA1*, two central regulators within the genetic susceptibility framework of gastric cancer (Kemp et al., 2026; Lobo et al., 2026). *CDH1* encodes E-cadherin, an indispensable mediator of epithelial cell–cell adhesion (Gall and Frampton, 2013). Loss-of-function variants, such as c.-160C>A and c.1531C>T, markedly suppress E-cadherin expression and serve as hallmark driver mutations in hereditary diffuse gastric cancer (Chen et al., 2011; Norero et al., 2019; Wu et al., 2025). The oncogenic potential of *CTNNA1* variants is second only to that of *CDH1*. Carriers of pathogenic *CTNNA1* mutations have a cumulative lifetime risk of 57% for developing gastric cancer by 80 years of age (Majewski et al., 2013; Coudert et al., 2022). Collectively, mutations in these genes represent pivotal molecular events underlying gastric cancer progression and may provide a foundation for early surveillance and targeted intervention strategies.

*ATM* is a key gene regulating DNA damage response and maintaining genomic stability. Studies indicate that *ATM* variants represent a notable hereditary risk factor for early-onset gastric cancer (Koebbe et al., 2025). Carriers of pathogenic *ATM* germline alterations develop gastric cancer at significantly younger ages than non-carriers, and the *ATM* rs189037 G > A polymorphism is associated with shorter overall survival among patients with gastric cancer (Helgason et al., 2015; Tao et al., 2020). Evidence suggests that carriers of pathogenic germline *BRCA1/2* mutations have an elevated risk of gastric cancer (Ichikawa et al., 2018; Avanesyan et al., 2020; Buckley et al., 2022). In particular, variants such as c.188 T>A, c.629>T, c.6637T>C, c.6922 A>T, and c.4427 A>G in *BRCA1/2* may facilitate optimized strategies for gastric cancer surveillance and precision treatment (Ichikawa et al., 2018). Additionally, the *MSH2* polymorphisms IVS10 + 12G>A and IVS12–6T>C may increase gastric cancer susceptibility in the Chinese population, especially when combined with lifestyle-related risk factors (Wang et al., 2012). A meta-analysis identified 11 genetic variants associated with gastric cancer risk, including seven SNPs (*MUC1* rs2070803, *MTX1* rs2075570, *PKLR* rs3762272, *PRKAA1* rs13361707, *TGFBR2* rs3087465, *CASP8* rs3834129, and *TNF* rs1799724) newly established as risk factors, further enriching the genetic susceptibility map of gastric cancer (Mocellin et al., 2015). However, the available evidence is derived primarily from small retrospective cohorts. Moreover, these genes predominantly drive tumorigenesis in breast and ovarian tissues and exhibit relatively weak specificity for gastric cancer; accordingly, they may be used only as secondary markers for risk stratification.

Furthermore, host genetic polymorphisms do not drive gastric carcinogenesis in isolation. Rather, they interact with pathogenic bacteria and epigenetic aberrations to synergistically increase gastric cancer risk. The pro-inflammatory cytokine interleukin (IL)-1β acts as a central mediator of gastric mucosal inflammation triggered by *H. pylori* colonization. Clinical studies have demonstrated that *IL-1β* polymorphisms exert synergistic oncogenic effects with *H. pylori* infection, markedly increasing individual susceptibility to gastric cancer (Gehmert et al., 2009; Hong et al., 2016). Functional evidence further indicates that IL-1β promotes aberrant promoter methylation, thereby facilitating tumorigenesis (Huang et al., 2016). Meanwhile, *H. pylori* infection predisposes the gastric mucosa to Cytosine-phosphate-Guanine (CpG) island hypermethylation and exacerbates global epigenetic dysregulation (Chan et al., 2007).

In summary, host genetic variants serve as promising molecular markers for gastric cancer risk stratification and early screening. However, these susceptibility genes differ substantially in oncogenic penetrance, strength of evidence, and clinical translational utility. Large-scale prospective cohort studies are warranted to quantify the independent pathogenic effects of different classes of genetic variants. In parallel, systematic investigations are needed to dissect the interactive crosstalk among host genetics, the microbiota, and epigenetic alterations, thereby facilitating the integration of genetic biomarkers into robust clinical risk-assessment models.

### 3.2 Epigenetic modifications

#### 3.2.1 DNA methylation

Aberrant hypermethylation of promoter CpG islands is a major mechanism underlying the inactivation of tumor suppressor genes. *H. pylori* infection, EBV infection, and various environmental stimuli can induce abnormal promoter methylation, thereby promoting the initiation and progression of gastric cancer (Qian et al., 2025). Studies have shown that aberrant methylation patterns in patients with gastric cancer are primarily characterized by global hypomethylation and CpG island hypermethylation, both of which may increase the risk of gastric mucosal carcinogenesis (Sudo et al., 2025). Hypermethylation of CpG islands within the promoters of multiple tumor suppressor genes, including *PRKCB*, *ID4*, and *CDKN2A*, has been widely reported, and these epigenetic abnormalities are closely associated with gastric cancer onset, invasion, and poor prognosis (Li et al., 2024; Luan et al., 2025; Nam et al., 2026). A deeper understanding of the tissue specificity and molecular mechanisms underlying methylation alterations in different genes may provide novel directions for the early detection and treatment of gastric cancer (Sepulveda et al., 2016). Studies have demonstrated that DNA methyltransferase (DNMT) inhibitors can reverse promoter hypermethylation and suppress the proliferative activity of gastric cancer cells (Luo et al., 2026). However, the clinical efficacy of these agents as monotherapy remains limited, and their optimal dosage has yet to be systematically established. Future studies should explore gastric mucosa-targeted delivery systems to enhance local drug accumulation at lesion sites. Alternatively, epigenetic therapies could be combined with gut microbiota modulation strategies to reverse the aberrant methylation patterns induced by pathogenic bacteria.

#### 3.2.2 Histone modifications

Histone modifications refer to covalent alterations of histone proteins, including acetylation, methylation, and ubiquitination, mediated by specific enzymes (Audia and Campbell, 2016). Histone acetylation is regulated by histone acetyltransferases (HATs) and histone deacetylases (HDACs). Multiple HDAC family members are aberrantly activated in gastric cancer and are capable of reshaping the tumor microenvironment while promoting tumor proliferation, invasion, and chemoresistance (Jiang Z. et al., 2019; Yang Z. et al., 2021; Zang et al., 2022). Consequently, HDACs have emerged as promising therapeutic targets in gastric cancer. Existing studies have demonstrated that targeted inhibition of HDACs not only suppresses gastric cancer progression but also exerts synergistic antitumor effects when combined with immunotherapy (Xu et al., 2018; Lin et al., 2023). In addition to histone acetylation, histone methylation plays a crucial role in gastric carcinogenesis. The methyltransferases SETD1A and G9A promote the proliferation and metastasis of gastric cancer cells by enhancing glycolysis and inducing DNA damage (Hu et al., 2018; Wu et al., 2020; Zhang et al., 2020). The histone demethylase retinoblastoma-binding protein 2 (RBP2), which is highly expressed in gastric tissue, is overexpressed in gastric cancer cells and activates the vascular endothelial growth factor signaling pathway, thereby accelerating tumor angiogenesis (Li et al., 2014). Specific inhibition of RBP2 induces cellular senescence and suppresses the continued proliferation of gastric cancer cells (Zeng et al., 2010).

#### 3.2.3 Non-coding RNAs

Non-coding RNAs (ncRNAs), including long non-coding RNAs (lncRNAs), microRNAs (miRNAs), and circular RNAs (circRNAs), play important roles in the progression of gastric cancer. Han et al. reviewed the major biological functions of ncRNAs in gastric cancer progression, including their involvement in chemotherapy resistance, tumor immune evasion, angiogenesis, and cellular metabolic reprogramming (Han et al., 2025). These findings highlight the potential of ncRNAs as molecular biomarkers and therapeutic targets for gastric cancer. In recent years, numerous dysregulated ncRNAs have been identified in gastric cancer tissues. Functionally, oncogenic ncRNAs are generally upregulated and promote cancer cell proliferation, invasion, and metastasis by activating canonical oncogenic signaling pathways or suppressing tumor suppressor genes. Representative examples include the lncRNAs CASC9, MIR181A1HG, and LINC00501; the miRNAs miR-17-5p, miR-215, miR-25, and miR-19a; and the circRNAs circMAT2B, circUBE2Q2, circCACTIN, and circNRIP1 (Deng et al., 2014; Li et al., 2015; Chen et al., 2016; Zhang L. et al., 2019; Zhang X. et al., 2019; Zhu et al., 2019; Liu et al., 2020; Li et al., 2021; Yang J. et al., 2021; Pei et al., 2023; Zhang J. et al., 2025). In contrast, tumor-suppressive ncRNAs are typically downregulated or absent in gastric cancer tissues. Restoration of their expression inhibits cancer cell growth and invasion while promoting apoptosis. Representative examples include miR-1254, miR-103a, miR-100-3p, miR-142-5p, and miR-1182, as well as the circRNAs circFAT1, circGRAMD1B, circRHOBTB3, circFAT1 (e2), and circ-CEP85L (Liang et al., 2015; Zhang et al., 2015; Dai et al., 2019; Jiang M. et al., 2019; Peng et al., 2019; Yan et al., 2019; Deng et al., 2020). The expression patterns, biological functions, and downstream targets of these ncRNAs are summarized in Table 1.

**Table 1:** Dysregulation of non-coding RNAs in gastric cancer.

Category	Name	Expression	Biological function	Target	References
Oncogenic ncRNAs	CASC9	Upregulation	Promote proliferation, invasion and metastasis	EGFR	Li et al. (2021)
	LINC00501	Upregulation	Promote proliferation, invasion, metastasis and tumor growth	STAT3	Pei et al. (2023)
	MIR181A1HG	Upregulation	Promote invasion, metastasis and epithelial–mesenchymal transition	ELAVL1	Zheng et al. (2025)
	miR-17-5p	Upregulation	Promote proliferation, migration, and invasion	EGR2	Chen et al. (2016)
	miR-215	Upregulation	Promote proliferation	RB1	Deng et al. (2014)
	miR-25	Upregulation	Promote proliferation, invasion, and migration	TOB1	Li et al. (2015)
	miR-19a	Upregulation	Promote proliferation, migration, and invasion	CUL5	Zhu et al. (2019)
	circMAT2B	Upregulation	Promote tumor growth	HIF-1α	Liu et al. (2020)
	circUBE2Q2	Upregulation	Promote proliferation, and migration	STAT3	Yang J. et al. (2021)
	circCACTIN	Upregulation	Promote proliferation, migration, invasion, and epithelial–mesenchymal transition	TGFBR1	Zhang L. et al. (2019)
	circNRIP1	Upregulation	Promote proliferation, migration, and invasion	AKT1	Zhang X. et al. (2019)
Tumor-suppressive ncRNAs	miR-1254	Downregulation	Suppress proliferation, migration, invasion, and epithelial–mesenchymal transition	Smurf1	Jiang M. et al. (2019)
	miR-103a	Downregulation	Suppress proliferation, migration, and invasion	c-Myb	Liang et al. (2015)
	miR-100-3p	Downregulation	Suppress migration, invasion, tumor growth, and promote apoptosis	BMPR2	Peng et al. (2019)
	miR-142-5p	Downregulation	Suppress migration and invasion	CYR61	Yan et al. (2019)
	miR-1182	Downregulation	Suppress proliferation and invasion	hTERT	Zhang et al. (2015)
	circGRAMD1B	Downregulation	Suppress proliferation, migration, and invasion	PTEN/p21	Dai et al. (2019)
	circRHOBTB3	Downregulation	Suppress tumor growth and tumor cell cycle progression	p21	Deng et al. (2020)
	circFAT1 (e2)	Downregulation	Suppress proliferation, invasion, and migration	YBX1	Fang et al. (2019)
	circ-CEP85L	Downregulation	Suppress proliferation and invasion	NFKBIA	Lu et al. (2020)

#### 3.2.4 RNA modifications

N6-methyladenosine (m6A) is an emerging field in the epigenetic regulation of gastric cancer (Ding et al., 2023). The m6A modification is dynamically regulated by “writer” proteins (e.g., METTL3, WTAP), “eraser” proteins (e.g., FTO), and “reader” proteins (e.g., YTHDF1, YTHDC2). METTL3 is overexpressed in gastric cancer tissues and promotes cell proliferation and metastasis by regulating the PBX homeobox 1 or Yes-associated protein 1 pathways. In contrast, METTL3 knockdown significantly inhibits gastric cancer cell proliferation, invasion, and metastasis *in vitro* (Zhou et al., 2021; Liu et al., 2022). Other “writer” proteins such as WTAP, KIAA1429, and METTL16, exhibit similar oncogenic functions and are strongly associated with poor prognosis (Miao et al., 2020; Wang et al., 2021; Yu et al., 2021). The “eraser” protein FTO promotes gastric cancer cell growth by regulating mitochondrial fission/fusion and cellular metabolism (Zhou Y. et al., 2022). Meanwhile, “reader” proteins such as YTHDC2 and YTHDF1 are overexpressed in gastric cancer and function as oncogenes that drive tumor initiation and progression (Chen et al., 2021; Yuan et al., 2022). Collectively, these findings underscore the pivotal role of the m6A regulatory network in gastric cancer.

## 4 Interactions among the microbiota, host genetics, and epigenetic modifications in gastric cancer

The development of gastric cancer is a complex, multifactorial process driven by environmental factors, the microbiota, and host genetics. While environmental factors play a crucial role in the early colonization and shaping of the gastrointestinal microbiota (Costello et al., 2012), studies using human twin cohorts and mouse models have confirmed that the host genetic background also significantly influences the regulation of microbial composition (McKnite et al., 2012; Goodrich et al., 2014). Importantly, microbiomes shaped by genetic or environmental factors—and their metabolites—can act as key environmental signals that induce epigenetic alterations in host cells, including DNA methylation, histone modifications, or RNA modifications (Pepke et al., 2024). These epigenetic changes subsequently regulate gene expression, ultimately influencing gastric cancer susceptibility and progression. Therefore, deciphering the interactive network among the microbiota, host genetics, and epigenetic modifications is essential for deepening our understanding of the mechanisms of gastric cancer pathogenesis.

### 4.1 Host genetic variation influencing microbiome composition in gastric cancer

Host genetic variation directly influences microbial colonization and abundance by shaping specific gastric microenvironments, thereby contributing to gastric cancer initiation. Extensive research has focused on interactions between polymorphisms in host immune-related genes and *H. pylori*. For example, genetic variants in pro-inflammatory cytokine genes, such as tumor necrosis factor-*α*, IL-10, and IL-1β, can enhance *H. pylori* virulence, induce gastric acid insufficiency and atrophic responses during infection, and significantly increase gastric cancer risk (El-Omar et al., 2003; Amieva and El-Omar, 2008; Ren et al., 2019). Beyond *H. pylori*, host genetic variation also regulates the composition of non-*H. pylori* microbiota. GWAS have revealed close associations between specific host genetic loci and bacterial genera. For instance, the *MUC1* rs4072037 C allele is associated with increased abundance of the human pathogen *Ochrobactrum*, which may exert carcinogenic effects through virulence characteristics shared with *H. pylori* (Kulkarni et al., 2014; Wang L. et al., 2020). Furthermore, RNA sequencing data from gastric cancer samples in public databases indicate that *DPH1* rs1131600 and *LRRC56* rs4963198 are associated with altered Corynebacterium abundance; *C9orf129* rs62572859, *FAT4* rs1014867, and *LRBA* rs1782360 are associated with changes in Lactobacillus abundance; and *ZC3H12D* rs61997220 correlates with alterations in both *Lactobacillus* and *Fusobacterium*. In addition, *PPP1R18* rs9262143, *SULT1C3* rs2219078, and *TRIM31* rs2523989 show the strongest associations with *Acinetobacter* abundance (Cavadas et al., 2020).

Although genetic polymorphisms correlate with microbiome composition, these associations exhibit substantial heterogeneity across different populations. Consequently, the identification of gastric cancer biomarkers based on host–microbiome interactions should account for geographic origin and the host’s ethnic genetic factors. Moreover, while most studies have reported correlations between host genetic variation and microbial composition, high-quality mechanistic studies elucidating how host genetics regulates the microbiota remain limited. Future research should integrate interventional experiments and multi-omics approaches to clarify the regulatory pathways linking host genetic variation and microbiota in gastric cancer.

### 4.2 Epigenetic alterations in gastric cancer induced by microbes and their metabolites

**Figure 2:**
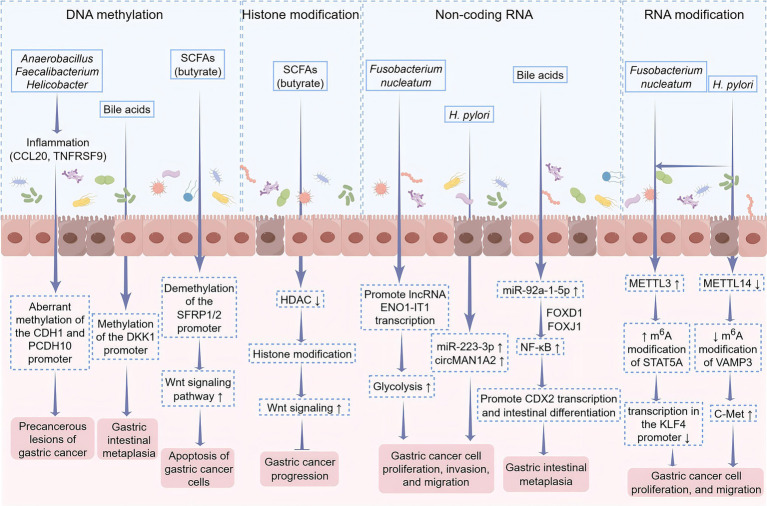
The microbiome and microbial metabolites modulate host epigenetics in gastric cancer. The microbiome and their derived metabolites are closely linked with host epigenetic modifications by influencing DNA methylation, histone modification, non-coding RNA, and RNA regulation. CCL20, C-C motif chemokine ligand 20; CDH1, Cadherin 1; C-Met, cellular-mesenchymal epithelial transition factor; DKK1, Dickkopf-related protein 1; ENO1-IT1, enolase1-intronic transcript 1; FOXD1, Forkhead box D1; FOXJ1, Forkhead box J1; HDAC, histone deacetylase; KLF4, Kruppel-like Factor 4; METTL3, methyltransferase 3; METTL14, methyltransferase 14; PCDH10, Protocadherin 10; SFRP1/2, Secreted Frizzled-Related Protein 1/2; STAT5A, signal transducer and activator of transcription 5A; TNFRSF9, tumor necrosis factor receptor superfamily member 9; VAMP3, vesicle associated membrane protein 3. Created with Figdraw.

Microbes and their metabolites are key regulators of the host epigenetic landscape. They regulate epigenetic processes such as DNA methylation, histone modifications, and non-coding RNAs (Fellows et al., 2018; Ansari et al., 2020; Haque et al., 2022) by providing epigenetic substrates or acting as enzyme modulators (Shock et al., 2021; Woo and Alenghat, 2022), thereby influencing the expression patterns of cancer-related genes (Figure 2). Elucidating the mechanisms by which microbes and their metabolites modulate epigenetic modifications will provide novel insights and therapeutic targets for the prevention and treatment of gastric cancer.

#### 4.2.1 Microbe-induced DNA methylation in gastric cancer

Exposure to commensal and pathogenic microorganisms during postnatal development induces distinct epigenetic and transcriptomic signatures in epithelial cells, highlighting the long-term effects of early-life microbial exposure on human health and disease (Cortese et al., 2016; Gensollen et al., 2016; Pan et al., 2018). Multiple clinical studies have identified significant associations between gastric cancer-associated dysbiosis and aberrant DNA methylation in the gastric mucosa, providing important clues linking the microbiota to epigenetic regulation during gastric tumorigenesis (Qian et al., 2025; Shimogama et al., 2025). Subsequent mechanistic studies have further supported a causal relationship between microbial colonization and epigenetic alterations. *H. pylori* and other gastric-associated microorganisms induce progressive hypermethylation of promoter CpG islands in cancer-related genes, and the accumulation of such aberrant methylation events increases gastric cancer risk (Maekita et al., 2006; Yan et al., 2023). By integrating analyses of human specimens with *in vitro* and *in vivo* experiments, Luan et al. demonstrated that *H. pylori* infection significantly increases methylation of the *ID4* promoter in both patients with gastric cancer and murine models. This epigenetic silencing abolishes the tumor-suppressive function of ID4, activates downstream oncogenic signaling pathways, and ultimately promotes gastric carcinogenesis (Luan et al., 2025).

Microbial metabolites act as critical molecular mediators linking gut dysbiosis to host epigenetic remodeling. BAs induce partial promoter methylation of *DKK1*, thereby facilitating the development of intestinal metaplasia, a well-established premalignant gastric lesion (Lu et al., 2020). Folate, B vitamins, and amino acids produced by probiotics serve as essential substrates for one-carbon metabolism and regulate the synthesis of S-adenosylmethionine (SAM), the primary cellular methyl donor. Alterations in microbial metabolite pools can therefore influence cellular SAM availability and reshape global DNA and histone methylation patterns in host epithelial cells (Rossi et al., 2011; Locasale, 2013; Oster et al., 2016; Woo and Alenghat, 2022). SCFAs, particularly butyrate, exert beneficial epigenetic effects by inducing demethylation of SFRP genes and histone modifications in gastric cancer cells, restoring gene expression and inhibiting the Wnt signaling pathway (Shin et al., 2012; Wang Q. et al., 2020). Collectively, these findings establish DNA methylation as a key mechanism through which the microbiota influences gastric cancer progression. Nevertheless, most available evidence is derived from observational human studies and therefore demonstrates association rather than causation. Definitive causal relationships between microbial exposure and aberrant DNA methylation require rigorous validation through dedicated *in vitro* and *in vivo* functional studies.

#### 4.2.2 Microbe-induced histone modifications in gastric cancer

Conventionally raised mice exhibit higher levels of histone acetylation, butyrylation, and propionylation than germ-free counterparts, confirming that commensal microbiota systematically reshape the host histone acylation landscape (Gates et al., 2024). Mechanistic studies have revealed that *γ*-glutamyl transferase secreted by *H. pylori* promotes histone hypermethylation in gastric epithelial cells, thereby hyperactivating Wnt signaling, reprogramming epithelial cell phenotypes, and accelerating gastric tumorigenesis (Jiang et al., 2024). As natural HDAC inhibitors, SCFAs are key regulators of histone acetylation (Li L. et al., 2022). SCFA-mediated HDAC inhibition modulates the acetylation of p70 S6 kinase and the phosphorylation of ribosomal protein S6, facilitating the differentiation of effector and regulatory T cells and thereby balancing immune tolerance and antitumor immune responses (Park et al., 2015). SCFAs also enhance glycolytic metabolism in immune cells and promote immunometabolic reprogramming through HAT-dependent histone acetylation (Wellen et al., 2009; Kim et al., 2016). In gastric cancer, histone acetylation levels are associated with tumor invasion and lymph node metastasis. By regulating histone acetylation, SCFAs reduce chromatin compaction, inhibit metastatic progression, and promote apoptosis of gastric cancer cells (Waldecker et al., 2008; Schizas et al., 2020).

#### 4.2.3 Microbe-induced non-coding RNAs in gastric cancer

Accumulating evidence indicates that diverse bacteria and viruses remodel ncRNA networks to regulate the proliferation, invasion, and metabolic reprogramming of gastric cancer cells. At the miRNA level, the *H. pylori* virulence factor CagA activates NF-κB signaling, leading to the upregulation of miR-223-3p, which targets and suppresses the tumor suppressor *ARID1A*, thereby promoting the initiation and progression of gastric cancer (Yang et al., 2018). EBV infection downregulates members of the miR-200 family, resulting in reduced E-cadherin expression and promoting EBV-associated gastric carcinogenesis (Shinozaki et al., 2010). During premalignant transformation, BAs upregulate miR-92a-1-5p, which suppresses *FOXD1* and subsequently induces CDX2 expression, thereby promoting intestinal metaplasia (Li et al., 2019). Regarding circRNAs, *H. pylori* infection increases circMAN1A2 expression, thereby enhancing the proliferation, migration, and invasion of gastric cancer cells, as demonstrated by *in vitro* functional studies (Guo et al., 2022). Additional studies have shown that intestinal dysbiosis promotes metastatic progression through circRNA–miRNA regulatory axes. In contrast, supplementation with probiotics such as *Bifidobacterium* can reverse these pathogenic ncRNA alterations and suppress tumor metastasis (Zhu et al., 2020). Collectively, these findings indicate that the microbiota orchestrates multiple stages of gastric cancer development through ncRNA-mediated regulation, and targeting the microbiota–ncRNA regulatory axis represents a promising therapeutic strategy for gastric cancer.

#### 4.2.4 Microbe-induced RNA modifications in gastric cancer

The crosstalk between the microbiota and RNA modifications, particularly m6A methylation, represents an emerging frontier in epigenetic research. Germ-free animal models provide compelling causal evidence that microbial colonization can globally remodel the host m6A epitranscriptome (Wang et al., 2019). *H. pylori* disrupts core components of the m6A regulatory machinery through multiple signaling pathways, thereby promoting the malignant transformation of gastric epithelial cells. Cellular and animal studies have demonstrated that *H. pylori* downregulates the m6A writer METTL14, reduces METTL14-dependent m6A deposition on *VAMP3* transcripts, and activates the LC3C-dependent c-Met recycling pathway, thereby sustaining gastric cancer progression (Cui et al., 2025). Furthermore, *H. pylori* suppresses the m6A reader YTHDF2 and hyperactivates NF-κB signaling, facilitating the malignant transformation of gastric mucosal cells (Wang D. et al., 2025). The virulence factor CagA markedly upregulates the m6A eraser FTO; elevated FTO expression removes m6A modifications and stabilizes heparin-binding EGF-like growth factor, ultimately triggering epithelial–mesenchymal transition in gastric cancer cells (He et al., 2025). Collectively, these findings demonstrate that *H. pylori* reshapes the host epitranscriptome by disrupting the balance among m6A writers, erasers, and readers, thereby promoting gastric cancer progression.

### 4.3 Impacts of epigenetic regulation on host genes and the microbiota

Epigenetic modifications regulate the transcription of gastric cancer-related genes and shape the malignant phenotypes of tumor cells. Epigenetic silencing of GATA transcription factors induces a GATA2/GATA6 expression switch in gastric tumor tissues, remodels the differentiation profile of gastric epithelial cells, and promotes the premalignant transformation of the gastric mucosa (Song et al., 2018). Beyond protein-coding genes, hypermethylation-mediated silencing of ncRNAs abolishes their inhibitory effects on downstream oncogenic targets, thereby exacerbating tumor malignancy. Specifically, epigenetic silencing of miR-124 disrupts its regulation of spermine oxidase (SMOX), facilitating SMOX-induced DNA damage and gastric carcinogenesis (Murray-Stewart et al., 2016). Similarly, epigenetic silencing of miR-1271 enhances oncogenic MEK1 and TEAD4 signaling, thereby accelerating the proliferation and invasion of gastric cancer cells (Lim et al., 2018). Moreover, epigenetic modulation of key cytokines reshapes the tumor microenvironment in ways that favor gastric cancer progression by regulating angiogenesis, metastasis, and chemoresistance (Reyes et al., 2024).

Beyond regulating host gene transcription, epigenetic modifications also function as upstream regulators of microbiota composition (Pepke et al., 2024). Evidence from studies of histone modifications strongly supports this concept. In *Drosophila*, the histone demethylase KDM5 maintains intestinal microbial homeostasis through immune signaling pathways; loss of KDM5 function disrupts commensal microbial communities and alters host physiological phenotypes (Chen et al., 2019). Alenghat et al. reported that HDAC3 senses signals from intestinal symbionts and regulates the expression of epithelial barrier genes to maintain microbial homeostasis. Intestine-specific deletion of HDAC3 reshapes the gut microbiota and increases susceptibility to intestinal inflammation (Alenghat et al., 2013). Host-derived fecal miRNAs also act as important epigenetic mediators that regulate microbial communities. These secreted miRNAs can enter intestinal bacteria and directly modulate the transcription of microbial metabolic genes, thereby reshaping the metabolic phenotypes of commensal microorganisms (Liu et al., 2016; Flanagan et al., 2025). Collectively, these findings demonstrate the bidirectional regulatory capacity of epigenetic mechanisms over both the microbiome and the host transcriptome.

Nevertheless, current studies in gastric cancer have largely established only correlative relationships between epigenetic abnormalities and alterations in the gastric microbiota, without elucidating the underlying mechanisms through which epigenetic modifications reshape microbial communities (Kim et al., 2024). Direct functional evidence demonstrating epigenetic remodeling of the gastric microbiome, therefore, remains a critical knowledge gap that warrants further investigation.

## 5 Potential advances in clinical applications

The tripartite network of host genetics, the microbiota and epigenetics offers multi-dimensional therapeutic targets for gastric cancer intervention. According to the regulatory crosstalk within this network, therapeutic strategies can be implemented separately at three levels: microbiota modulation, epigenetic reprogramming, and stratification based on host genetic background. Combinatorial interventions targeting multiple nodes are also theoretically feasible. Nevertheless, most strategies remain in the preclinical or early exploratory stage, and their translational potential requires rigorous clinical validation.

Microbiota-targeted interventions, including probiotics, prebiotics, and postbiotics, have shown promising efficacy in preclinical studies. Probiotic formulations containing *Bifidobacterium infantis*, *Lactobacillus acidophilus*, and *Enterococcus faecalis* can reshape microbial diversity, modulate host immunity, and suppress inflammatory responses in patients with gastric cancer (Zheng et al., 2019). Metabolites produced by probiotics may increase histone acetylation, reactivate silenced tumor suppressor genes, and reverse aberrant promoter hypermethylation, thereby delaying gastric cancer progression (Waldecker et al., 2008; He et al., 2023). Collectively, the antitumor activity of probiotics is largely mediated by SCFAs, which inhibit HDAC activity and restore normal DNA methylation patterns. Inulin-type fructans and polyphenol-based prebiotics enrich beneficial commensal microorganisms and inhibit the adhesion and colonization of *H. pylori*, thereby suppressing gastric tumorigenesis through multiple mechanisms (Roberfroid et al., 2010; Khan et al., 2018; Ma and Chen, 2020). As bioactive microbial metabolites, postbiotics exert diverse effects, including maintaining microbial homeostasis, strengthening epithelial barrier integrity, and modulating host immune responses (Cotter et al., 2005; Jaye et al., 2022). For example, certain postbiotics can co-aggregate with *H. pylori* and impair its mucosal adhesion and virulence (Hua et al., 2025). *In vitro* studies have further demonstrated that butyrate synergizes with dexamethasone to downregulate the oncogene *TNS4* (Eladwy et al., 2024). However, these observations are mostly derived from cell and animal experiments. Clinical evidence confirming that probiotics exert antitumor effects via epigenetic reprogramming in human gastric cancer patients is still absent. Notably, probiotic efficacy exhibits strong strain specificity. Metabolite profiles differ drastically across species and even among different strains of the same species, which creates major obstacles for developing standardized treatment protocols.

The reversible nature of epigenetic modifications makes them attractive therapeutic targets. Preclinical *in vitro* and *in vivo* studies have demonstrated that the HDAC inhibitor chidamide sensitizes gastric cancer cells to 5-fluorouracil, offering a potential strategy for overcoming chemoresistance (Zhang X. et al., 2025). Another HDAC inhibitor, entinostat, downregulates human epidermal growth factor receptor 2 (HER2) expression in gastric cancer cells, providing a rationale for both monotherapy and combination therapy in HER2-amplified gastric cancer (Zenz et al., 2025). The m6A demethylase ALKBH5 suppresses the translation of uncapped WRAP53 transcripts in an m6A-dependent manner, thereby inhibiting gastric cancer proliferation and metastasis (Zheng et al., 2025). In addition, essential oil extracted from *Origanum onites L*. exhibits antitumor activity against gastric cancer by modulating promoter methylation and histone modifications of tumor suppressor genes (Sogutlu et al., 2025). Despite these encouraging preclinical findings, translational evidence supporting epigenetic-targeted therapies remains limited, and their clinical utility requires further validation. Furthermore, no clinical trials have yet validated the efficacy and safety of combining epigenetic agents with microbiota-targeted therapies.

Host genetic variants are key determinants of gastric cancer susceptibility. High-risk individuals carrying germline mutations with high penetrance would benefit from regular endoscopic surveillance and preventive intervention. It remains unclear how host genotypes shape individual responses to microbiota and epigenetic therapies. The clinical value of microbiota-targeted and epigenetic interventions in individuals carrying pathogenic genetic variants remains uncertain. Future studies should determine whether genetic background influences patient responsiveness to microbiota-targeted or epigenetic therapies, thereby providing a foundation for genetically stratified precision medicine approaches.

In summary, targeting only a single node of this network rarely achieves durable anti-tumor effects. Multi-node combinatorial therapies may produce synergistic benefits by simultaneously correcting microbial dysbiosis and reversing epigenetic aberrations. However, the optimal administration sequence, dosage combinations and eligible patient populations for combination treatment have not been systematically characterized. Importantly, live biotherapeutics such as fecal microbiota transplantation carry substantial infectious risks among immunocompromised gastric cancer patients. Strict criteria for donor screening and recipient selection are urgently needed to improve safety. Phage therapy for gastric cancer is still in its infancy. Rapid emergence of bacterial phage resistance and poor phage stability under gastric acidic conditions constitute major translational barriers. Well-designed clinical trials are warranted to systematically evaluate the safety and efficacy of these emerging treatment modalities.

## 6 Conclusions and prospects

Gastric carcinogenesis is a complex, multi-factorial process orchestrated by an interconnected regulatory network involving host genetics, the microbiota, and epigenetic modifications. Microorganisms and their metabolites induce epigenetic abnormalities that silence tumor suppressor genes or activate oncogenes. Host genetic variants can promote microbial dysbiosis and amplify microbe-driven epigenetic damage. In turn, aberrant epigenetic remodeling reshapes microbial composition and rewires host transcriptional programs. These reciprocal interactions form a self-reinforcing feedback loop that progressively increases the risk of malignant transformation. Nevertheless, several critical knowledge gaps remain.

First, many studies fail to adequately account for confounding factors, such as geographic variation and environmental exposures, that influence microbial communities and may compromise the reliability of study conclusions. Cross-cohort integrative analyses should therefore carefully adjust for population, dietary, and regional heterogeneity to minimize bias. Second, most studies report only associations among microbial dysbiosis, genetic variation, and epigenetic abnormalities, without establishing definitive causal relationships. Gene-edited cell lines and germ-free mouse colonization models could help elucidate the mechanistic links between microorganisms and epigenetic remodeling. In parallel, large-scale prospective cohort studies are needed to determine how host genetic polymorphisms influence microbial composition and interact with epigenetic alterations. Third, direct *in vivo* evidence supporting epigenetic regulation of the gastric mucosal microbiota and host transcription remains limited. Tissue-specific conditional knockout mouse models combined with 16S rRNA sequencing and multi-omics analyses may provide valuable insights into how epigenetic alterations reshape gastric microbial communities and host gene expression. Finally, current microbiota-targeted and epigenetic interventions have limited clinical applicability, and their efficacy and safety remain insufficiently validated. Future therapeutic strategies should integrate genomic, microbiomic, and epigenomic information to develop combination therapies tailored to individual molecular profiles.

In summary, interpreting gastric carcinogenesis through the framework of the host genetics–microbiota–epigenetics network overcomes the limitations of traditional single-factor mechanistic models. Future studies focusing on causal validation, epigenetic regulation of the microbiota, and stratified personalized interventions will facilitate the development of more precise preventive and therapeutic strategies for gastric cancer.

## Glossary

ATM: ataxia telangiectasia mutated
BAs: bile acids
BRCA1/2: breast cancer susceptibility genes 1/2
CDH1: cadherin 1
circRNAs: circular RNAs
CpG: cytosine-phosphate-Guanine
CTNNA1: catenin alpha 1
DNMT: DNA methyltransferases
EBV: Epstein–Barr virus
GWAS: genome-wide association studies
HATs: histone acetyltransferases
HDACs: histone deacetylases
HER2: human epidermal growth factor receptor 2
lncRNAs: long non-coding RNAs
LPS: lipopolysaccharide
m6A: N6-methyladenosine
miRNAs: microRNAs
MSH2: MutS protein homolog 2
ncRNAs: non-coding RNAs
PGVs: pathogenic germline variants
RBP2: retinoblastoma-binding protein 2
SAM: S-adenosylmethionine
SCFAs: short-chain fatty acids
SNPs: single-nucleotide polymorphisms
SMOX: spermine oxidase
TMAO: trimethylamine N-oxide
TP53: tumor protein p53
